# Supplementary material for: Ethnic Issues and Disparities in Inflammatory Bowel Diseases: What Can We Learn from the Arab Population in Israel?
Source: J Pers Med. 2023 Jun 17;13(6):1008. doi: 10.3390/jpm13061008 (PMC10301587; doi:10.3390/jpm13061008)
Supplement: Supplementary file 1 [file jpm-13-01008-s001.zip › jpm-2421161-supplementary.pdf]

Table S1. Comorbidities among the Crohn's disease subgroups .

Comorbidities: Crohn's Disease	Arabs 1263 (%)	Jews 11625 (%)	*p*-value
CIHD	172 (13.6)	1613 (13.9)	0.802
COPD	115 (9.1)	1007 (8.7)	0.596
Asthma	186 (14.7)	1864 (16)	0.228
Chronic Kidney disease	114 (9)	1157 (10)	0.294
Hypertension	301 (23.8)	3231 (27.8)	0.003
Diabetes Mellitus	154 (12.2)	998 (8.6)	<0.001
Dyslipidemia	371 (29.4)	3692 (31.8)	0.083
Obesity	358 (28.3)	2511 (21.6)	<0.001
Fatty liver	79 (6.3)	990 (8.5)	0.006
CVA	30 (2.1)	238 (2)	0.438
Cirrhosis	22 (1.7)	123 (1.1)	0.029
Dementia	17 (1.3)	345 (3)	0.001
Vitamin B12 Deficiency	31 (2.5)	232 (2)	0.273
Folic acid deficiency	314 (24.9)	3627 (31.2)	<0.001
Iron anemia deficiency	590 (46.7)	5442 (46.8)	0.947
Vitamin D deficiency	251 (19.9)	2786 (24)	0.001
Clostridium difficile infection	34 (2.7)	277 (2.4)	0.496
Pneumonia	333 (26.4)	3259 (28)	0.209
Depression	160 (12.7)	2266 (19.5)	<0.001
Male infertility	83 (6.6)	292 (2.5)	<0.001
Female infertility	78 (6.2)	898 (7.7)	0.048
Osteoporosis	87 (6.9)	888 (7.6)	0.338
Colon cancer	14 (1.1)	195 (1.7)	0.128
Rectal cancer	1 (0.08)	46 (0.4)	0.076
Lymphoma-non-Hodgkin	9 (0.7)	98 (0.8)	0.628
Lymphoma—Hodgkin	5 (0.4)	30 (0.3)	0.371
Melanoma	2 (0.2)	168 (1.4)	<0.001
Basal cell carcinoma	25 (2)	1257 (10.8)	<0.001
Small bowel cancer	1 (0.1)	29 (0.2)	0.233

CIHD=Chronic Ischemic Heart Disease, COPD=Chronic Obstructive Pulmonary Disease, CVA=Cerebrovascular Accident

Table S2. Comorbidities among ulcerative colitis patients.

Ulcerative colitis Comorbidities	Arabs n = 1461 (%)	Jews 10920 (%)	*p*-value
CIHD	203 (13.9)	2019 (18.5)	<0.001
COPD	115 (7.9)	1018 (9.3)	0.071
Asthma	218 (14.9)	1574 (14.4)	0.605
Chronic Kidney Disease	107 (7.3)	1251 (11.5)	<0.001
Hypertension	360 (24.6)	3842 (35)	<0.001
Diabetes Mellitus	174 (11.9)	1310 (12)	0.924
Dyslipidemia	470 (32.2)	4649 (42.6)	<0.001
Obesity	424 (29)	2509 (23)	<0.001
Fatty liver	61 (4.2)	925 (8.5)	<0.001
CVA	31 (2.1)	275 (2.5)	0.359
Cirrhosis	14 (1)	136 (1.2)	0.346

Dementia	28 (1.9)	449 (4.1)	<0.001
Vitamin B12 Deficiency	19 (1.3)	160 (1.5)	0.620
Folic acid deficiency	263 (18)	2638 (24.2)	<0.001
Iron anemia deficiency	621 (42.5)	4221 (38.7)	0.005
Vitamin D deficiency	285 (19.5)	2481 (22.7)	0.006
Clostridium difficile infection	30 (2.1)	301 (2.8)	0.118
Pneumonia	393 (26.9)	3104 (28.4)	0.224
Depression	172 (11.8)	2112 (19.3)	<0.001
Male infertility	76 (5.2)	235 (2.2)	<0.001
Female infertility	116 (7.9)	742 (6.8)	0.106
Osteoporosis	84 (5.7)	853 (7.8)	0.005
Colon cancer	20 (1.4)	236 (2.2)	0.046
Rectal cancer	4 (0.3)	78 (0.7)	0.051
Lymphoma-non-Hodgkin	5 (0.3)	81 (0.7)	0.084
Lymphoma—Hodgkin	1 (0.07)	21 (0.2)	0.291
Melanoma	6 (0.4)	199 (1.8)	<0.001
Basal cell carcinoma	31 (2.1)	1442 (13.2)	<0.001
Small bowel cancer	6 (0.4)	40 (0.4)	793

CIHD=Chronic Ischemic Heart Disease, COPD=Chronic Obstructive Pulmonary Disease, CVA=Cerebrovascular Accident
